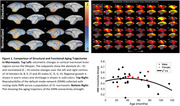# Resting State Functional Connectivity Assessment of Healthy Aging Trajectories in the Marmoset Brain

**DOI:** 10.1002/alz.090994

**Published:** 2025-01-09

**Authors:** Rebecca Bhik‐Ghanie, Diego Szczupak, Bei Zhang, Daniel Papoti, Vinicius Campos, Lauren R Dubberley, T Kevin Hitchens, Fang‐Cheng Yeh, Lauren K Hayrynen Schaeffer, Gregory W Carter, Stacey J Sukoff Rizzo, David J Schaeffer, Afonso C Silva

**Affiliations:** ^1^ University of Pittsburgh School of Medicine, Pittsburgh, PA USA; ^2^ Universidade Federal do ABC, Sao Bernardo do Campo, Sao Paulo Brazil; ^3^ Universidade de Sao Paulo, Sao Carlos, Sao Paulo Brazil; ^4^ The Jackson Laboratory, Bar Harbor, ME USA

## Abstract

**Background:**

The common marmoset (Callithrix jacchus) is an important animal model in neuroscience and neurological diseases, presenting primate‐specific evolutionary features such as an expanded frontal cortex. We established a new consortium with funding support from the National Institute on Aging to generate, characterize, and validate MArmosets as Research MOdels of AD (MARMO‐AD). This consortium develops and studies gene‐edited marmoset models carrying genetic risk for AD, comparing them against wild‐type aging marmosets from birth throughout their lifespan, using non‐invasive longitudinal assessments. Here, we aim to characterize healthy aging trajectories by investigating their resting state functional connectivity in a population of marmosets.

**Method:**

We imaged a cohort of 25 marmosets (17 males, 8 females) across the lifespan (8 to 150 months) using a dedicated 9.4T/30cm Bruker MRI scanner. The animals were acclimated to restrainers and head‐fixation helmets and imaged awake. EPI images (500 µm isotropic) were acquired for 1 hour and 20 minutes, yielding 2400 whole‐brain volumes. The images were pre‐processed for fMRI using AFNI and FSL. For each run, the first ten time points were removed for magnetization to reach a steady state. The images were despiked, and spatially aligned. Slice timing was corrected, and phase‐encoding distortion was corrected using FSL. Subsequently, brain images were registered to the Marmoset Brain Mapping V3 template, and brain‐wide connectomics were calculated using the GRETNA toolbox.

**Result:**

Our group has shown through structural investigations (voxel‐based morphometry and white matter tractography) that several cortical clusters are affected by aging (Figure 1, Top Left). Here, with resting state fMRI, we show that many brain regions that change size with aging also show accompanying changes in their functional connectivity strength (Figure 1, Right). Integrating resting‐state fMRI with structural investigations will allow us to understand how these affected structures work together in a dynamic network.

**Conclusion:**

Our work is the first to thoroughly describe the changes in resting state functional connectivity in the marmoset brain during normal aging, a valuable model for AD. This research will establish normative baselines for changes in marmoset brain connectivity with aging to evaluate our genetically engineered marmoset models of AD.